# Transcriptome sequencing of *Tessaratoma papillosa* antennae to identify and analyze expression patterns of putative olfaction genes

**DOI:** 10.1038/s41598-017-03306-7

**Published:** 2017-06-08

**Authors:** Zhong-Zhen Wu, Meng-Qiu Qu, Xin-Hua Pu, Yang Cui, Wan-Yu Xiao, Hong-Xia Zhao, Shu-Ying Bin, Jin-Tian Lin

**Affiliations:** 1 Institute for Management of Invasive Alien Species, 314 Yingdong teaching building, Zhongkai University of Agriculture and Engineering, Guangzhou, 510225 P. R. China; 2 Guangdong Institute of Applied Biological Resources, Guangzhou, 510260 P. R. China

## Abstract

Studies on insect olfaction have increased our understanding of insect’s chemosensory system and chemical ecology, and have improved pest control strategies based on insect behavior. In this study, we assembled the antennal transcriptomes of the lychee giant stink bug, *Tessaratoma papillosa*, by using next generation sequencing to identify the major olfaction gene families in this species. In total, 59 odorant receptors, 14 ionotropic receptors (8 antennal IRs), and 33 odorant binding proteins (28 classic OBPs and 5 plus-C OBPs) were identified from the male and female antennal transcriptomes. Analyses of tissue expression profiles revealed that all 59 OR transcripts, 2 of the 8 antennal IRs, and 6 of the 33 OBPs were primarily expressed in the antennae, suggesting their putative role in olfaction. The sex-biased expression patterns of these antenna-predominant genes suggested that they may have important functions in the reproductive behavior of these insects. This is the first report that provides a comprehensive resource to future studies on olfaction in the lychee giant stink bug.

## Introduction

The main function of insect olfaction is to detect odor molecules in the environment to guide insects towards food sources, mating partners, and oviposition sites as well as to avoid predators and other dangers. Studies on insect olfaction have provided insights into the chemosensory biology and chemical ecology of insects, and improved the management strategies for behavior-based pest control. In insects, primary odorant reception occurs in the antennae that contain olfactory receptor neurons (ORNs)^1^. At the molecular level, the peripheral olfactory proteins including odorant receptors (ORs), ionotropic receptors (IRs), and odorant-binding proteins (OBPs) have been shown to interact with specific sets of ligands and to play active roles in odorant detection^2, 3^.

Insect ORs are members of a large group of proteins belonging to the novel 7-transmembrane domain protein family. ORs are present within the dendritic membrane of the olfactory sensory neurons (OSNs)^4–6^. These ORs form ligand-gated ion channels with highly divergent OR subunits (ORx), which together with the well-conserved OR co-receptor (Orco), impart ligand specificity^7, 8^. IRs belong to an ancient family of insect chemoreceptors that are related to the ionotropic glutamate receptors (iGluR) and are divided into two subfamilies, the conserved “antennal IRs” involved in olfaction and species-specific “divergent IRs,” which are detected in *Drosophila* gustatory organs rather than the olfactory organs and function as candidate gustatory and pheromone receptors^9–18^. In addition, two IR co-receptors, IR8a and IR25a, are broadly expressed and analogous to the Orco, play an essential role in tuning IR sensory cilia targeting and IR-based sensory channels^18^. OBPs, on the other hand, are water-soluble globular proteins containing six conserved cysteine residues paired into three interlocked disulfide bridges and participate in the solubilization and transfer of odorants across the sensillum lymph. The OBPs are known to contribute to the sensitivity of the olfactory system^19–22^. However, none of the olfactory gene families have been characterized in the lychee giant stink bug, *Tessaratoma papillosa* (Drury) (Hemiptera: Tessaratomidae).

*T*. *papillosa* primarily damages lychee and longan, and can also infest other economically important fruit trees such as oranges, pomegranates, pomelos and cannas under certain conditions. This pest species is not only widespread in South China but is also widely distributed in South Asia and the neighboring regions (http://www.cabi.org/isc/datasheet/53273). The stink bug *T*. *papillosa* belongs to the Pentatomidae family that can release their foul-smelling fluids in response to disturbance or aggression^23–25^. Previous studies have shown that these glandular secretions primarily function in defense against predators, and contain alarm pheromones, attractant pheromones, sex pheromones, etc. Therefore, these cues may provide valuable information to monitor and control *T*. *papillosa* by using pheromones. More importantly, targeting the peripheral olfactory proteins has helped to design and predict the best possible semiochemical to be used in management strategies for behavior-based pest control^12, 26–28^.

In this study, we used RNA-sequencing to identify putative OR, IR, and OBP transcripts in the adult *T*. *papillosa* antennae. We also investigated their phylogenetic relationships with known olfaction-related proteins from other insect species, and examined OR, IR, and OBP gene transcription patterns in *T*. *papillosa*. The findings of this study will provide a basis for the functional characterization of putative olfaction genes in *T*. *papillosa*. Enhancing our understanding of the molecular mechanisms of olfaction in *T*. *papillosa* could lead to the identification of potential new targets for olfactory disruption and the development of safe pest control strategies.

## Results

### Sequencing, assembly and functional annotation

**Figure 1:**
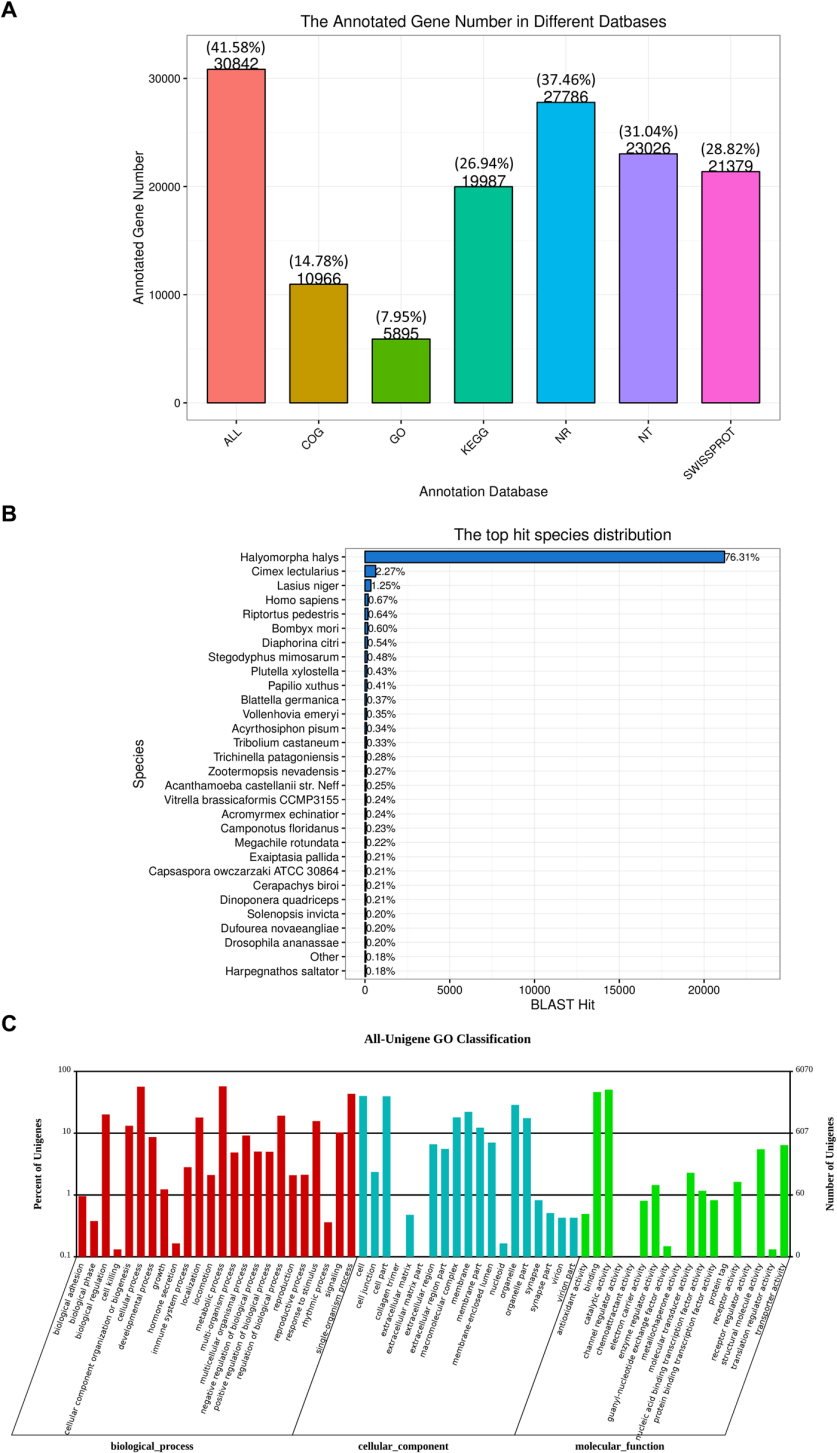
(**A**) Unigenes annotated via different databases. (**B**) Percentage of top hits from other insect species homologous to *T*. *papillosa* transcripts. Transcripts from *T*. *papillosa* were searched by BLASTx against the non-redundant protein database with a cutoff E-value of 10^−5^. (**C**) Gene ontology (GO) classification of *T*. *papillosa* transcripts using Blast2GO. One unigene was annotated to more than one GO term.

Through the Illumina HiSeq 4000 RNA-Seq strategy, high quality transcriptome data were obtained. In total, 48,627,008 clean reads were obtained from adult female antennae and 48,634,546 clean reads from adult male antennae, with a mean length of 150 base pairs (bp). Trinity assembly of both male and female antennal transcriptomes generated 74,183 unigenes with a mean length of 1,095 bp and a N50 length of 2,342 bp (Supplementary Table S1). Among these, 30,842 (41.58%) unigenes were annotated at least to one of the databases; 27,786 (37.46%) unigenes were annotated by the NCBI-Nr database, 23,026 (31.04%) unigenes by the NCBI-Nt database, 21,379 (28.82%) by SwissPort, 19,987 (26.94%) by KEGG, 5,895 (7.95%) by GO, 10,966 (14.78%) by the COG database (Fig. 1A). BLASTx homology searches against the NCBI-Nr database showed that *T*. *papillosa* antennal transcriptomes shared the highest homology (76.31%) with sequences from *Halyomorpha halys*, the brown marmorated stink bug, followed by sequences from *Cimex lectularius*, the bed bug (2.27%) (Fig. 1B). Gene Ontology (GO) annotation was used to classify the *T*. *papillosa* antennal transcriptome unigenes into functional groups according to the GO terms. In the molecular function GO category, the most abundant transcripts in the antenna were associated with binding and catalytic functions. In the biological process category, the transcripts were mostly associated with cellular, metabolic, and single-organism processes. In the cellular component category, cell, cell part, and organelle were the most represented (Fig. 1C).

### Major olfactory-related proteins in *T*. *papillosa* antennae

In this study, the major olfactory-related proteins including ORs (59 transcripts), IRs (14 transcripts), and OBPs (33 transcripts) were identified in *T*. *papillosa* antennal transcriptomes of both sexes. Information of the identified ORs, IRs, OBPs, and reference genes including the unigene sequences, lengths, and the best BLASTx hits, predicted protein domains and transcript abundance are listed in Supplementary Dataset File.

#### Candidate odorant receptors

**Figure 2:**
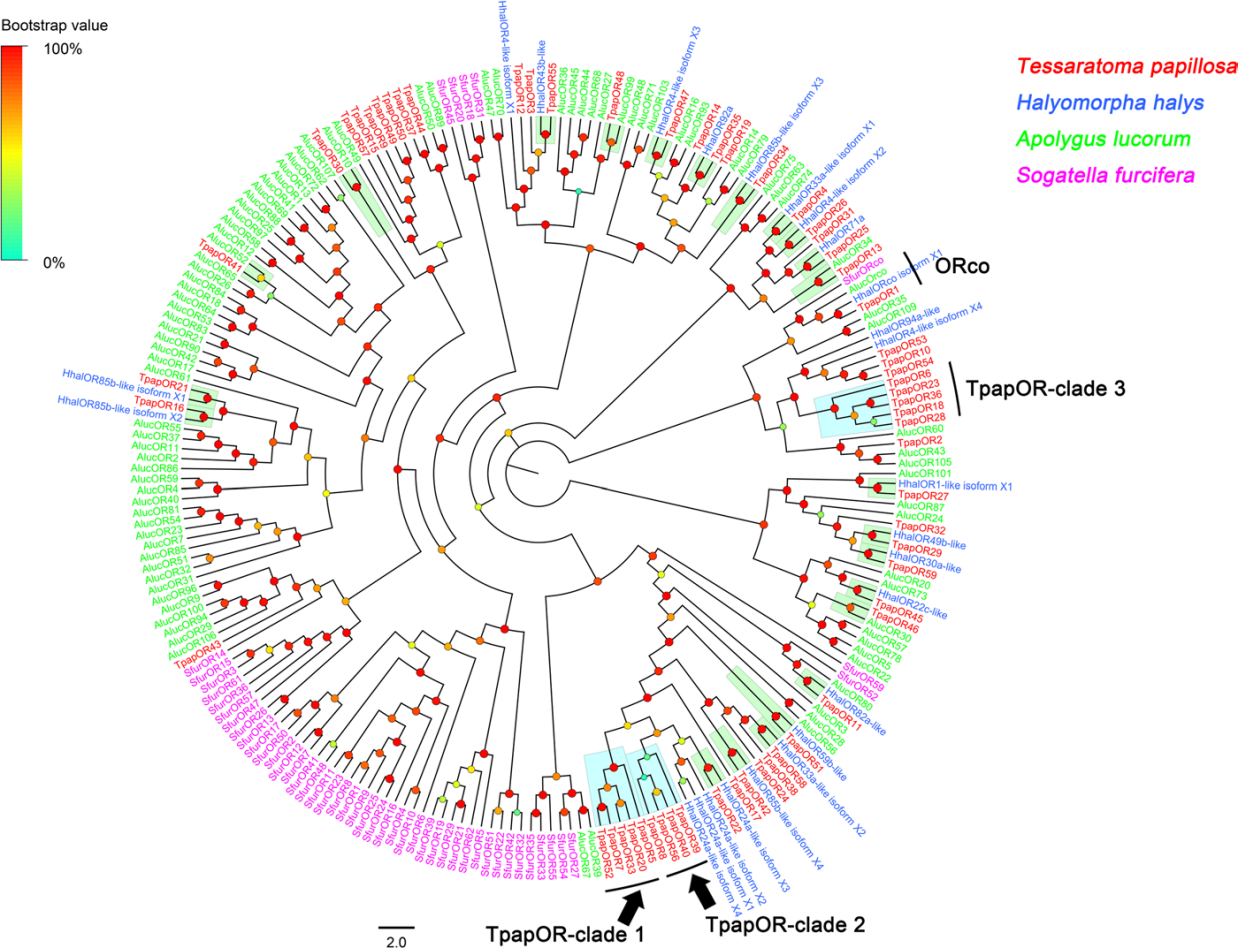
Phylogenetic analysis of putative *T*. *papillosa* ORs with other hemipteran ORs. The dendrogram was generated by FastTree2 (JTT substitution model). Species abbreviations: Tpap, *Tessaratoma papillosa*; Aluc, *Apolygus lucorum*; Hhal, *Halyomorpha halys*; Sfur, *Sogatella furcifera*. Amino acid sequences used for tree construction are shown in Supplementary Table S2. Branch support (circles at the branch nodes) was estimated using an approximate likelihood ratio test based on the scale indicated at the top left. Bars indicate branch lengths in proportion to amino acid substitutions per site.

**Figure 3:**
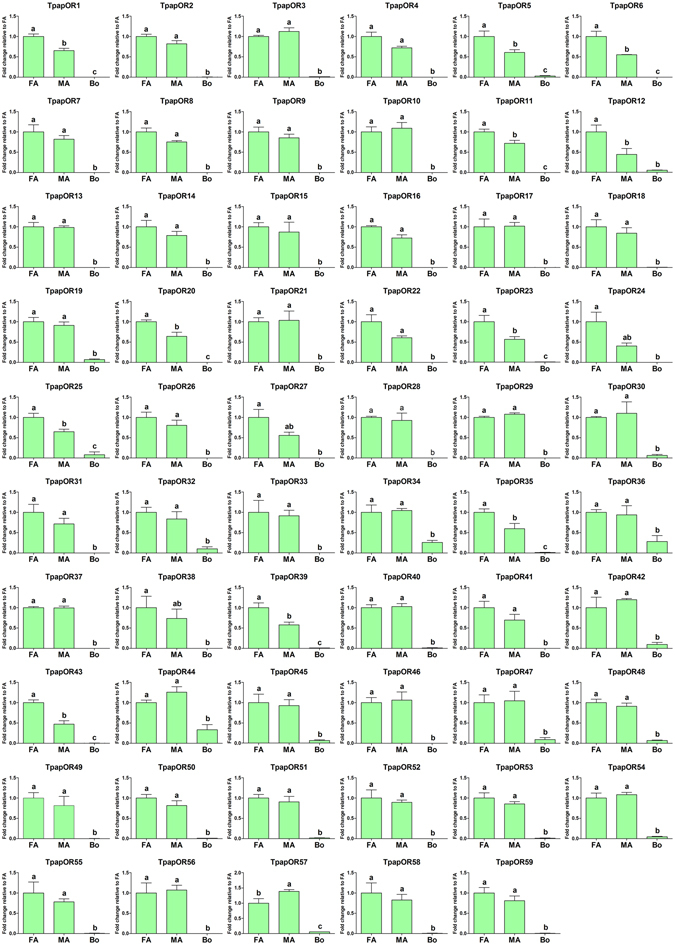
Relative expression levels of putative *T*. *papillosa* ORs in the female and male antennae, and other body parts. Abbreviations: FA, female antennae; MA, female antennae; Bo, other body parts (the pooled tissue mixture of proboscis, stink gland, midgut, foreleg tarsus, and wing). The expression levels were estimated using the 2^−ΔΔCT^ method. The relative expression level is indicated as mean ± SE (n = 4). Standard error is represented by the error bar, and different letters indicate statistically significant difference between tissues (p < 0.05, ANOVA, HSD).

Bioinformatics analysis of the *T*. *papillosa* antennal transcriptomes identified 59 transcripts as candidate ORs, which have several putative transmembrane domains (TMDs). Among these, 45 were found to likely contain full-length open reading frames (ORF), encoding proteins with more than 313 amino acid residues, and 4 to 8 transmembrane domains. The partial TpapORs identified in our transcriptome were predicted to have 2 to 6 transmembrane domains (Supplementary Dataset File). With the exception of Orco, the predicted ORs shared 26–84% amino acid sequence identity with homologous ORs in other species, which was evident from our BLASTx analysis. A phylogenetic analysis was conducted using a data set containing all 59 ORs in *T*. *papillosa* together with other hemipteran ORs including the green plant bug *Apolygus lucorum*, the brown marmorated stink bug *Halyomorpha halys* and the white-backed planthopper *Sogatella furcifera* (Fig. 2). In the phylogenetic tree, the highly conserved co-receptor, Orco, shared 88–99% amino acid sequence identity and clustered with orthologous proteins from four other hemipteran species (Fig. 2). A large number of *T*. *papillosa* ORs could be assigned to putative orthologs in two Hemiptera species; 17 TpapORs clustered with HhalORs and 5 TpapORs clustered with AlucORs, suggesting that these hemipteran ORs may possess certain common olfactory functions. Thus, three monophyletic subgroups were evident (TpapOR-clade 1 to TpapOR-clade 3), indicating that these perform species-specific functions (Fig. 2). The expression profiles of TpapORs were characterized using qPCR, and the results revealed that all TpapORs displayed predominant expression in antennae. Although we did not identify apparent sex-specific genes in these *T*. *papillosa* ORs, TpapOR57 was significantly upregulated in the male and nine ORs including TpapOR1, 6, 11, 12, 20, 23, 25, 39 and 43 were significantly upregulated in the female antennae (Fig. 3).

#### Candidate ionotropic receptors

**Figure 4:**
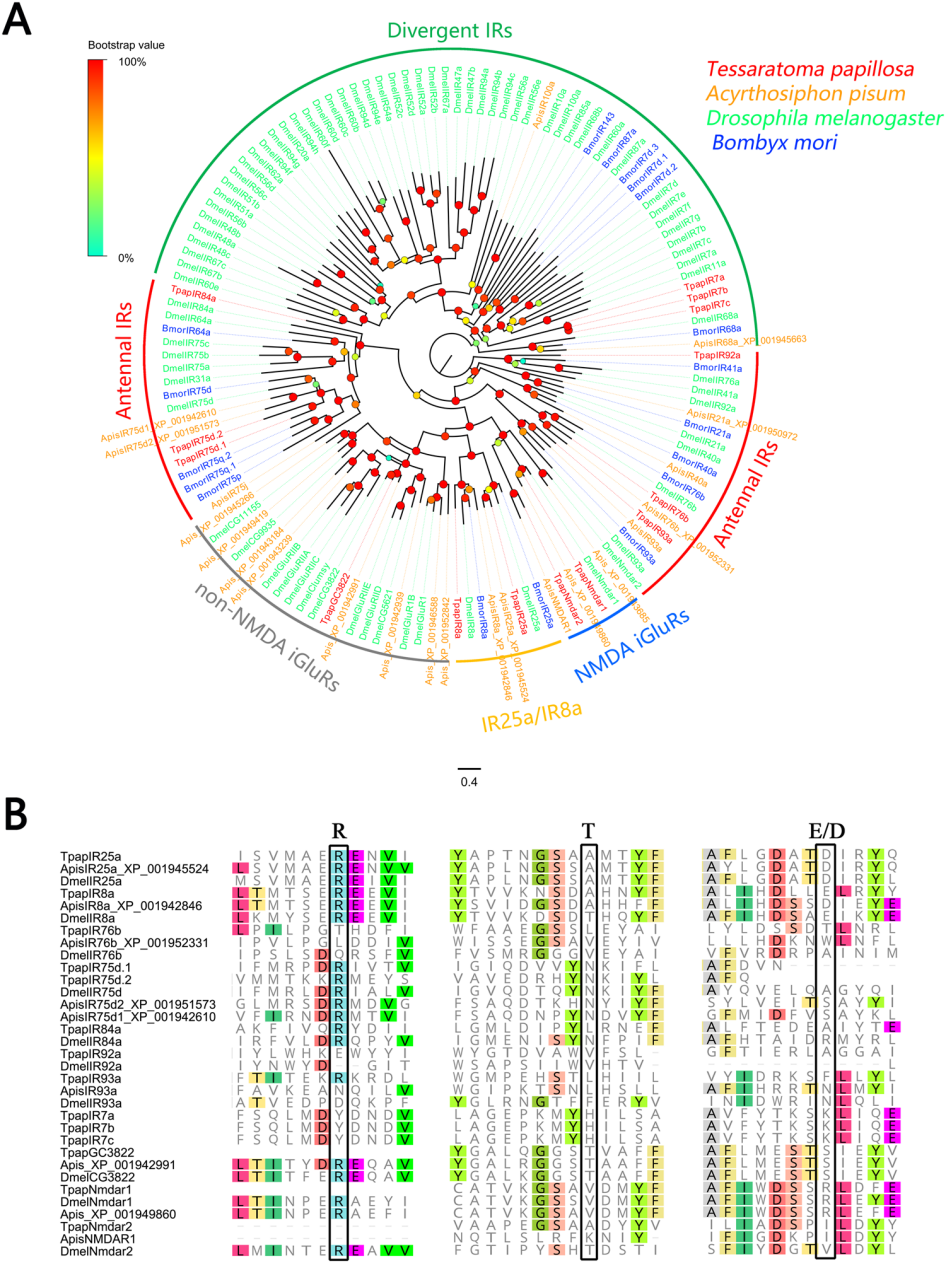
(**A**) Phylogenetic analysis of putative *T*. *papillosa* iGluRs/IRs in with other insect iGluRs/IRs. The dendrogram was generated by FastTree2 (JTT substitution model). Species abbreviations: Tpap, *Tessaratoma papillosa*; Apis, *Acyrthosiphon pisum*; Bmor, *Bombyx mori*; Dmel, *Drosophila melanogaster*. Amino acid sequences used to construct the tree are shown in Supplementary Table S2. Branch support (circles at the branch nodes) was estimated using an approximate likelihood ratio test based on the scale indicated at the top left. Bars indicate branch lengths in proportion to amino acid substitutions per site. (**B**) Excerpts from the amino acids alignment showing the predicted iGluRs/IRs binding domains.

**Figure 5:**
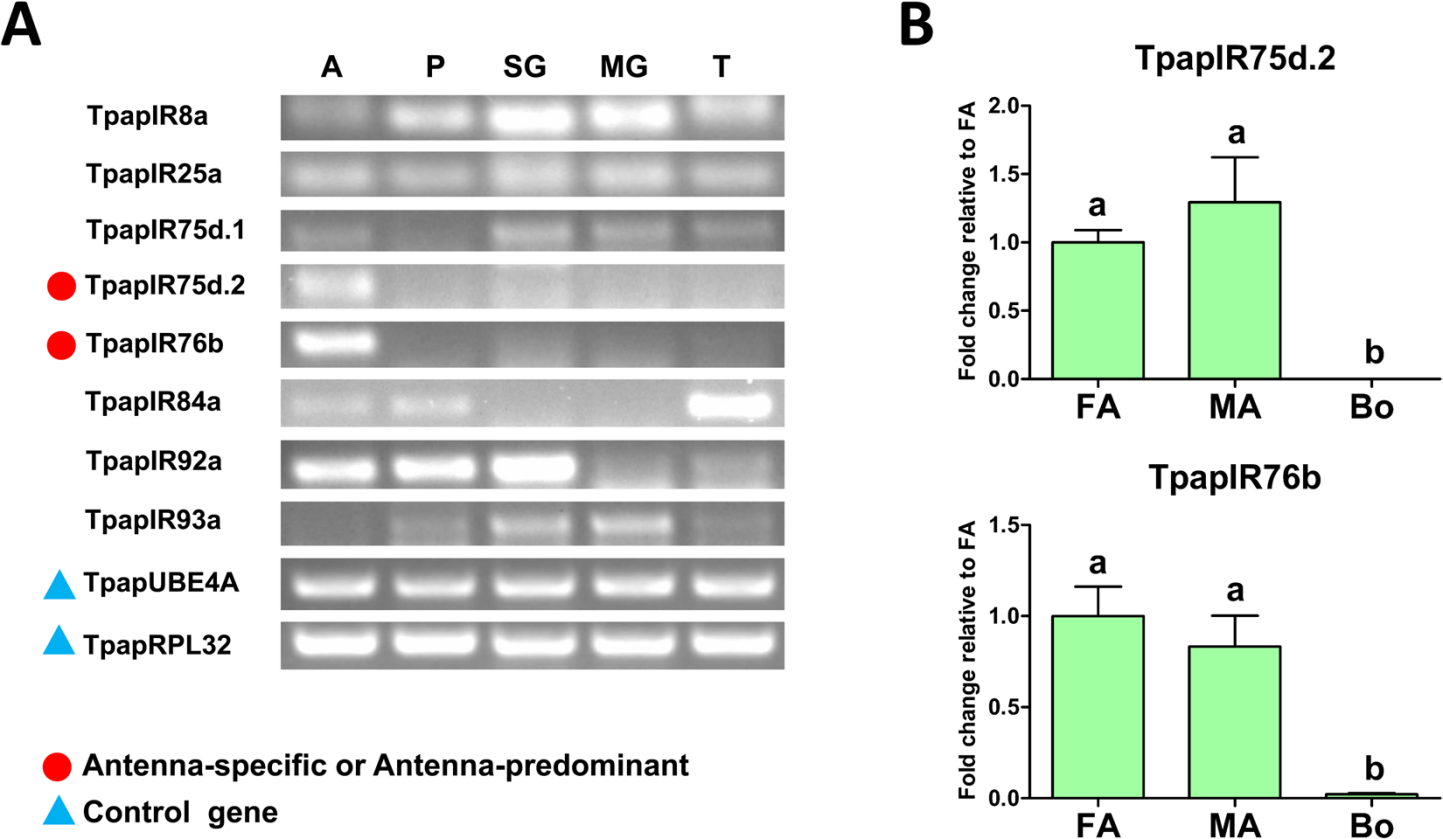
(**A**) Transcriptional profiles of putative *T*. *papillosa* iGluRs/IRs in different body parts as determined by semi-quantitative RT-PCR. Red dots represent iGluRs/IRs that are upregulated in the antenna. Two reference genes labeled with blue triangle, ubiquitin conjugation factor E4 A (TpapUBE4A) and 60 S ribosomal protein L32 (TpapRPL32), were used as internal references to test the integrity of each cDNA template. Abbreviations: A: antenna; P, proboscis; SG, stink gland; MG, midgut; T, tarsus. (**B**) Relative expression levels of antenna-predominant iGluRs/IRs in the female and male antennae, and other body parts. Abbreviations: FA, female antennae; MA, female antennae; Bo, other body parts (the pooled tissue mixture of proboscis, stink gland, midgut, foreleg tarsus, and wing). The expression levels were estimated using the 2^−ΔΔCT^ method. Relative expression level is indicated as mean ± SE (n = 4). Standard error is represented by the error bar, and different letters indicate statistically significant difference between tissues (p < 0.05, ANOVA, HSD).

We identified 14 transcripts encoding candidate iGluRs/IRs from the *T*. *papillosa* antennal transcriptomes. These were predicted to encode ligand-gated cation channels. Of these, seven candidates likely contain full-length ORFs (TpapIR8a, 25a, 76b, 92a, 7a, 7b and 7c) with three transmembrane domains. Because of the relatively high conservation among IRs^18^, we obtained orthologous genes from other insects including *Acyrthosiphon pisum*, *D*. *melanogaster* and *Bombyx mori* and assigned them to five phylogenetic groups; N-Methyl-D-aspartic acid (NMDA) iGluRs, non-NMDA iGluRs, IR25a/IR8a, divergent IRs and antennal IRs. We found that 6 TpapIRs clustered with the conserved antennal IR subfamily, which is thought to play a role in odor detection (Fig. 4A). Among these antennal IRs, only two IR co-receptors (TpapIR25a and TpapIR8a) retained all residues characteristic to glutamate binding domains (R, T and E/D)^17^, while in other IRs one or several were absent (Fig. 4B). Semi-quantitative RT-PCR showed that only two antennal IRs (TpapIR75d.2 and TpapIR76b) were present predominantly in *T*. *papillosa* antennae (Fig. 5A), and exhibited no difference in gene expression among the sexes by qPCR analyses (Fig. 5B).

#### Candidate odorant binding proteins

**Figure 6:**
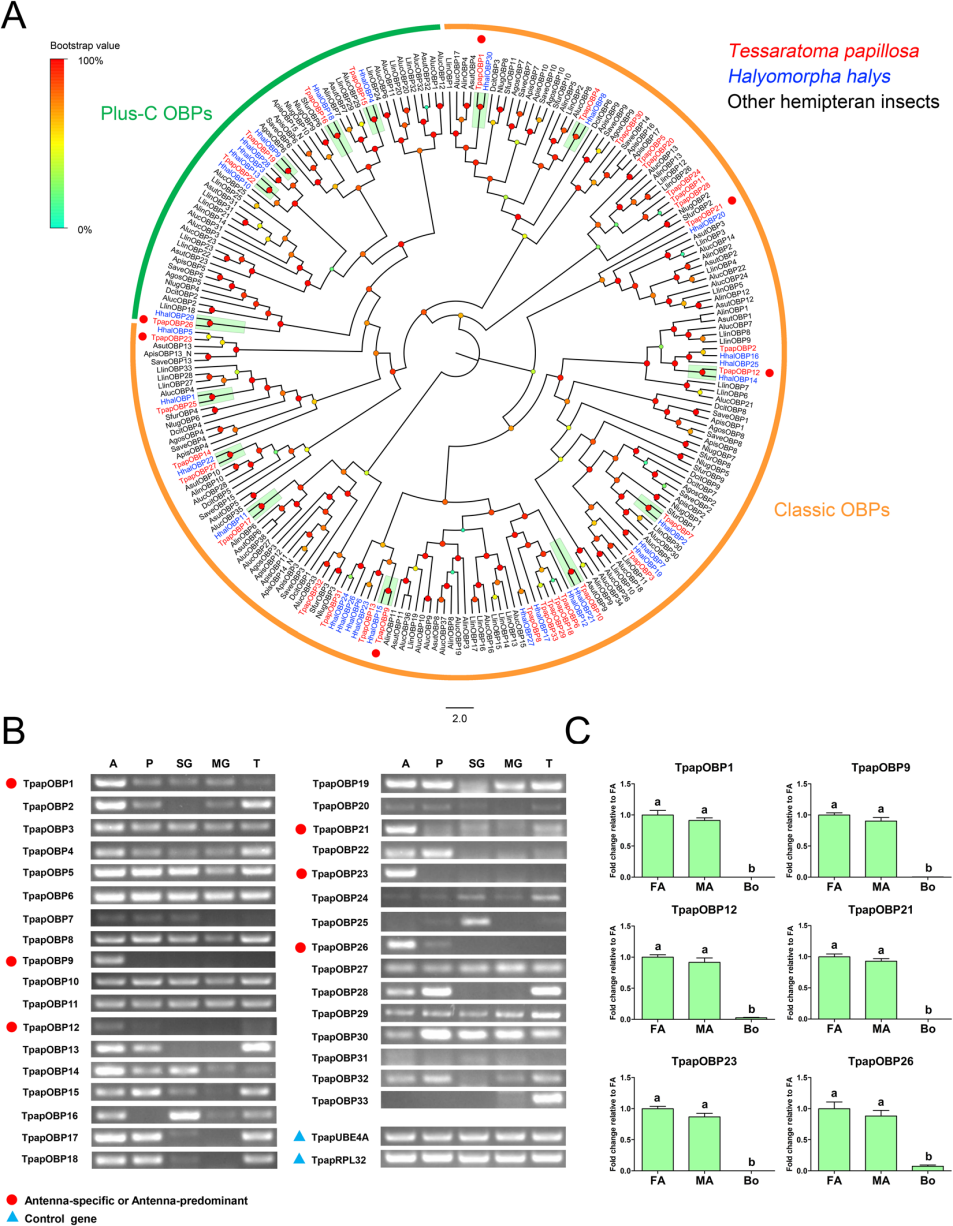
(**A**) Phylogenetic analysis of putative *T*. *papillosa* OBPs with other hemipteran OBPs. Plus-C OBPs from *T*. *papillosa* and other putative hemipterans form a clade labeled in dark green. Labeled in orange are classic OBPs from *T*. *papillosa* and other putative hemipterans. Species abbreviations: Tpap, *Tessaratoma papillosa*; Hhal, *Halyomorpha halys*; Llin, *Lygus lineolaris*; Aluc, *Apolygus lucorum*. Alin, *Adelphocoris lineolatus*, Asut, *Adelphocoris suturalis*, Save, *Sitobion avenae*, Apis, *Acyrthosiphon pisum*, Agos, *Aphis gossypii*, Nlug, *Nilaparvata lugens*, Sfur, *Sogatella furcifera*, Dcit, *Diaphorina citri*. Amino acid sequences used to construct the tree are shown in Supplementary Table S2. (**B**) Transcriptional profiles of putative *T*. *papillosa* OBPs in different body parts determined by semi-quantitative RT-PCR. The OBPs that are upregulated in the antennae are labeled with red dots. Two reference genes labeled with blue triangle, ubiquitin conjugation factor E4 A (TpapUBE4A) and 60 S ribosomal protein L32 (TpapRPL32), were used as internal references to test the integrity of each cDNA templates. Abbreviations: A: antenna; P, proboscis; SG, stink gland; MG, midgut; T, tarsus. (**C**) Relative expression levels of antenna-predominant OBPs in the female and male antennae, and other body parts. Abbreviations: FA, female antennae; MA, female antennae; Bo, other body parts. Expression levels were estimated using the 2^−ΔΔCT^ method. The relative expression level is indicated as mean ± SE (n = 4). Standard error is represented by the error bar, and different letters indicate statistically significant difference between tissues (p < 0.05, ANOVA, HSD).

In total, we identified 33 candidate OBP transcripts. All TpapOBP transcripts except five (TpapOBP2, 18, 29, 30 and 32) contain full-length ORFs with signal peptides. According to the primary protein structure of insect OBPs^22^, 28 TpapOBPs were classified as classic OBPs with the conserved six-cysteines residues, and five (TpapOBP15, 16, 19, 22 and 26) were Plus-C OBPs with additional conserved Cys (C1a, C1b, C1c, C6a, C6b and C6c) plus one proline after the C6a (see Supplementary Figure S1). Based on the number of cysteine residues, phylogenetic analysis of TpapOBPs with OBPs from five plant bugs and other hemipterans (including two aphids, two planthoppers, and one psyllid) showed that a group of Plus-C OBPs in *T*. *papillosa* clustered with homologous Plus-C OBPs from other hemipterans to form a unique clade, and the remainder Classic OBPs clustered with homologous Classic OBPs. A significant number of TpapOBPs were orthologous to *H*. *halys* OBPs with a high bootstrap value (Fig. 6A). The TpapOBP transcripts displayed different patterns of tissue distribution and abundance (Fig. 6B). Six transcripts (TpapOBP1, 9, 12, 21, 23 and 26) were present predominantly in the antennae. TpapOBP22 was abundant in the antennae, but was also present in the proboscis. Similarly, TpapOBP25 was abundant in scent glands, while TpapOBP33 was abundant in the tarsi; both OBPs were also present in other tissues. Other OBP transcripts were present in all or several body parts. In addition, sex-biased expression was not detected in the six antenna-predominant OBPs analyzed by qPCR (Fig. 6C).

## Discussion

The molecular basis of olfaction in hemipterans is relatively poorly understood compared to those of dipterans and lepidopterans. In this study, we investigated the transcriptome of the lychee giant stink bug, *T*. *papillosa*, to better understand odor detection in hemipterans. This bug releases disagreeable odor volatiles when disturbed or aggravated, and information of the olfactory processes of this bug will be useful for effective pest management. Computational prediction of the interactions between peripheral olfactory proteins and odor molecules has significantly advanced the identification of new activators and inhibitors^12, 26, 27^. Thus, the identification of gene families involved in odorant reception and detection represent a valuable genomic resource for future population control strategies.

Function prediction of the male and female *T*. *papillosa* transcripts through GO assignment generated results similar to those reported previously for dipteran^29–31^, coleopteran^32–34^, hymenopteran^35, 36^, lepidopteran^37–40^ and other hemipteran antennal transcriptomes^41^. Such comparison with other insect orders suggested a certain level of conservation among the molecular component in the peripheral olfactory system.

In the *T*. *papillosa* antennal transcriptomes, a total of 59 candidate OR proteins were identified. This total number is much lower when compared with 110 antennal ORs reported in the green plant bug, *Apolygus lucorum* ^42^. It is very likely that not all OR genes have been identified in this research. However, we believe the majority of ORs have been identified in this species based on the over number of reads and the quality of the sequences. Assuming that most OR genes have been identified in this research, the smaller number of ORs in *T*. *papillosa* may indicate specialization of ORs as an adaptation to specialized ecological niches. Indeed, *A*. *lucorum* has a broad range of host plants^43^, while *T*. *papillosa* has relatively narrow host plant range (http://www.cabi.org/isc/datasheet/53273)^44^. Additionally, antennal expression of OR genes was validated with qPCR. By comparing the sex-biased antennal expression, we found that TpapOR57 has male-biased expression and may be associated with detecting sex pheromones or male specific behaviors. On the other hand, TpapOR1, 6, 11, 12, 20, 23, 25, 39 and 43 had female-biased expression, indicating their likely involvement in female specific behaviors i.e., finding plant hosts for oviposition or other female-specific functions. Generally, the OR co-receptor expression level is about the same in both male and female antennae, but the OR co-receptor in *T*. *papillosa* was expressed at a higher level in female antennae. This might be related to the distribution pattern and amounts of olfactory sensilla in this species. These analyses revealed a likely correlation was well as distinct differences between male and female *T*. *papillosa* in the distribution pattern and amounts. For example, sensilla cavity is specifically present in female antennae^45^.

IRs have been mostly studied in *Drosophila*, and can be categorized into two groups; the olfaction oriented “antennal” IRs and the gustatory, divergent species-specific IRs^11, 18^. The antennal IRs confer response to diverse volatiles including acids and amines, food odor, and DEET repellency^9, 12, 15, 16, 46, 47^. In this study, 8 antennal IR candidates including two co-receptors, (IR8a and IR25a) were found in the *T*. *papillosa* antennal transcriptomes. This number is lower than that expressed in the antennae of adult *D*. *melanogaster* (18 antennal IRs)^48^. We postulate that reduction of IRs in *T*. *papillosa* may reflect to adaptation of this insect species to an environment with low “semiochemical diversity”. Additionally, among our identified *T*. *papillosa* antennal IRs, only two (TpapIR75d.2 and TpapIR76b) presented a clear antenna-predominant expression, while other members were not restricted to the antennae. In the *Drosophila* olfactory system, the ionotropic receptor IR76b is co-expressed with the specific IR (IR41a) to mediate long-range attraction to the odor^9^. Thus, IR76b in *T*. *papillosa*, could likely play a similar role in olfactory perception. However, because *T*. *papillosa* antennal IRs do not have some key amino acids in the glutamate binding domains, various non-coreceptor IR candidates and TpapIR76b, other functions for these antennal IRs cannot be ruled out.

Polyphagous stink bugs have a broad range of host plants, with some overlap among them. The numbers of putative OBP-encoding transcripts identified in *T*. *papillosa* (33 OBPs) are similar to the number reported in the antennal transcriptome of three plant bugs, *Lygus lineolaris* (32 OBPs)^49^, *H*. *halys* (30 OBPs)^41^ and *Apolygus lucorum* (38 OBPs)^50^ but more than the number expressed in the antennae of other Heteropteras (Pentatomidae) *Adelphocoris lineolatus* (14 OBPs)^51^, and *Adelphocoris suturalis* (16 OBPs)^52^. This difference could be potentially attributed to their specialized ecology. OBPs are not restricted to the olfactory tissues and have been proposed to be involved in other non-sensory functions^53–60^. Here, we found that most TpapOBPs were distributed in all examined body parts and only six candidates (2 Plus-C OBPs and 4 Classic OBPs) were present predominantly in the antennae, suggesting that they may likely contribute to the sensitivity of insect olfactory system^61–63^. Recently, the alarm pheromone stimulus-induced effect resulted in up-regulating the expression levels of twenty-one *H*. *halys* OBPs in the antennae, suggesting that multiple OBPs may respond to this pheromone^41^. Addition of antenna-predominant candidates to the existing olfactory related gene pool could provide clues to future studies on the functional characterization of *T*. *papillosa*.

## Conclusion

This study reports the first antennal transcriptome analysis of an important fruit pest, *T*. *papillosa*. The transcriptome analysis reported here provides valuable insight into the molecular mechanisms of olfaction in the lychee giant stink bug. Putative olfaction genes including ORs, IRs and OBPs were identified, and their transcriptional profiles were investigated to confirm roles of these genes. Our results greatly improve the gene inventory for *T*. *papillosa* and provide a valuable resource for future analysis on stink bug olfaction.

## Methods

### Insect rearing

A mass of *T*. *papillosa* eggs were collected from an experimental field with lychee trees in the Guangdong Provincial Zhongkai University of Agriculture and Engineering at Guangzhou, China (23.10°E, 113.27°N). Lychee leaves with eggs were shipped in an artificial climate box for mass rearing at 28 °C ± 1 °C, 80% relative humidity and a 12 h:12 h light:dark photoperiod. Hatched nymphs were reared on fresh lychee leaves until adults emerged. Adults (20-days-old) were segregated based on sex according to the external genitalia.

### RNA isolation, sequencing, *de novo* assembly, and annotation

Antennae from male and female bugs were dissected from 20-days-old adults (n = 50) and frozen separately in liquid nitrogen. Then, they were powdered and total RNA was isolated using the TRIzol reagent (Invitrogen, USA). RNA concentration was determined with a ND-2000 spectrophotometer (Thermo Scientific, USA), and RNA integrity value (RIN) was confirmed on the Bioanalyzer 2100 system (Agilent Technologies, USA). cDNA library was prepared using Illumina’s sample preparation instructions (Illumina, San Diego, CA). The library was then sequenced using the Illumina HiSeq4000 system (Illumina Inc. San Diego, CA) to obtain paired-end reads. The raw sequence transcriptome data from the female and male antennae libraries have been submitted to the NCBI Short Read Archive (SRA) database as BioProject Accession Number SRP077039.

The raw reads were first preprocessed by filtering for unknown (poly-N) or low-quality sequences and adaptor sequences. *De novo* assembly of the filtered transcriptome data (clean reads) was performed using the Trinity pipeline (version r2013-02-25) with default parameters^64^. Functional annotations of the unigenes were performed using BLASTXx and comparing the transcripts to diverse protein databases including NCBI-Nr, NCBI-Nt, Pfam, KOG/COG, Swiss-prot, KEGG and GO, according to the highest sequence similarity.

### Gene identification

To comprehensively identify OR, IR, and OBP transcripts, both BLAST2GO annotation and tBLASTn searches (Geneious software) were adopted. Queries for tBLASTn searches were amino acid sequences of known insect species collected from NCBI with keywords such as “odorant receptor AND insecta”, “ionotropic receptor OR ionotropic glutamate receptor AND insecta”, “odorant-binding protein AND insecta”. The open reading frames (ORFs) were predicted in the ORF finder tool at NCBI, and translated to amino acid sequence in Geneious (version 9.1.3.). In addition, presence of definitive domains (e.g. transmembrane domains, signal peptides, secondary structures, etc.) in ORs, IRs, and OBPs was further predicted by queries against InterPro using the InterProScan tool plug-in in Geneious (version 9.1.3.)^65^.

### Sequence alignment and phylogenetic analysis

For structural comparison among IRs and OBPs, alignments of the respective amino acid sequences were carried out using the E-INS-I strategy in MAFFT in Geneious (version 9.1.3.)^66^. Phylogenetic analysis of the putative *T*. *papillosa* olfaction genes was performed in conjunction with other insect amino acid sequences from previously published data (Supplementary Table S2). The putative amino acid sequences from *T*. *papillosa* ORs, IRs, and OBPs (without the signal peptides) were aligned with the MAFFT alignment tool (E-INS-I parameter) plug-in in Geneious (version 9.1.3.)^66^, and the dendrograms were then calculated using FastTree2 (JTT substitution model)^67^, and visualized with FigTree (http://tree.bio.ed.ac.uk/software/figtree). Node support for the phylogenetic tree was assessed using the bootstrap method with 1000 bootstrap replicates.

### Tissue expression analysis

Total RNA from the analyzed tissues was extracted with TRIzol reagent (Invitrogen, USA) and treated with DNase I (TAKARA, China) to remove trace amounts of genomic DNA. cDNA was synthesized from total RNA using PrimeScript RT reagent Kit (TAKARA, China). Specific primer pairs were designed in Primer3web (version 4.0.0) (http://primer3.ut.ee/) (Supplementary Table S3). Semi-quantitative RT-PCR was employed to investigate and compare the expression of olfaction-related genes in different tissues including antennae, proboscis, stink gland, midgut and foreleg tarsus (n = 10 each). PCR was performed under the following conditions: 95 °C for 2 min, followed by 35 cycles of 95 °C for 30 sec, 56 °C for 30 sec, 72 °C for 1 min, and a final extension for 10 min at 72 °C. Each semi-quantitative RT-PCR was repeated two times using two independently isolated RNA samples. PCR amplification products were separated on a 1.5% agarose gel. Two reference genes, ubiquitin conjugation factor E4 A (TpapUBE4A) and 60 S ribosomal protein L32 (TpapRPL32) from *T*. *papillosa* antennal transcriptomes were used as the control^68^.

qPCR was used to quantify expression levels of the antenna-predominant candidates among IRs, OBPs, and ORs. First, RNA extraction and cDNA synthesis were performed in samples including 20 male or female antennae each and other body parts (the pooled tissue mixture of proboscis, stink glands, midguts, foreleg tarsus, and wings, n = 10 each). qPCR analysis was conducted on a LightCycler 480 system (Roche Applied Science) with a SYBR Premix ExTaq kit (TAKARA, China). The cycling parameters were as follows: 95 °C for 15 min, followed by 40 cycles of 95 °C for 10 sec and 60 °C for 32 sec. Then, the PCR products were heated to 95 °C for 15 sec, cooled to 60 °C for 1 min, heated to 95 °C for 30 sec and cooled to 60 °C for 15 sec to measure the dissociation curves. Negative controls without template were included in each experiment. To check reproducibility, each qPCR reaction was performed using three technical replicates and four biological replicates. Relative quantification was performed with the 2^−ΔΔCT^ method^69^. All data were normalized to reference genes levels from the same tissue samples and the relative fold change in different tissues was calculated with the transcript level of the female antennae as calibrator. The comparative analyses of each target gene among different tissues were determined using a one-way nested analysis of variance (ANOVA) followed by Tukey’s honest significance difference (HSD) test using Prism 6.0 (GraphPad Software, CA). Values are presented as mean ± SE.

## Electronic supplementary material

Supplementary Information

Supplementary Dataset
