# Supplementary material for: Transcriptome sequencing of Tessaratoma papillosa antennae to identify and analyze expression patterns of putative olfaction genes
Source: Sci Rep. 2017 Jun 8;7:3070. doi: 10.1038/s41598-017-03306-7 (PMC5465196; doi:10.1038/s41598-017-03306-7)
Supplement: Supplementary file 1 — Supplementary Information [file 41598_2017_3306_MOESM1_ESM.pdf]

Supplementary Information for

Transcriptome sequencing of *Tessaratoma papillosa* antennae to identify and analyze expression patterns of putative olfaction genes

Zhong-Zhen Wu[1], Meng-Qiu Qu[1], Xin-Hua Pu[1], Yang Cui[1], Wan-Yu Xiao[1], Hong-Xia Zhao[2], Shu-Ying Bin[1], Jin-Tian Lin[1*]

[1]Institute for Management of Invasive Alien Species, 314 Yingdong teaching building, Zhongkai University of Agriculture and Engineering, Guangzhou 510225, PR China

[2]Guangdong Institute of Applied Biological Resources, Guangzhou 510260, PR China

[*]Corresponding author

Email addresses:

Zhong-Zhen Wu: zhongzhen_wu@163.com

Meng-Qiu Qu: qumengqiu@163.com

Xin-Hua Pu: pu123xh@163.com

Yang Cui: muttcy@163.com

Wan-Yu Xiao: yuwanxiao22@126.com

Hong-Xia Zhao: Hxzh110@126.com

Shu-Ying Bin: binsuying@163.com

Jin-Tian Lin: linjtian@163.com

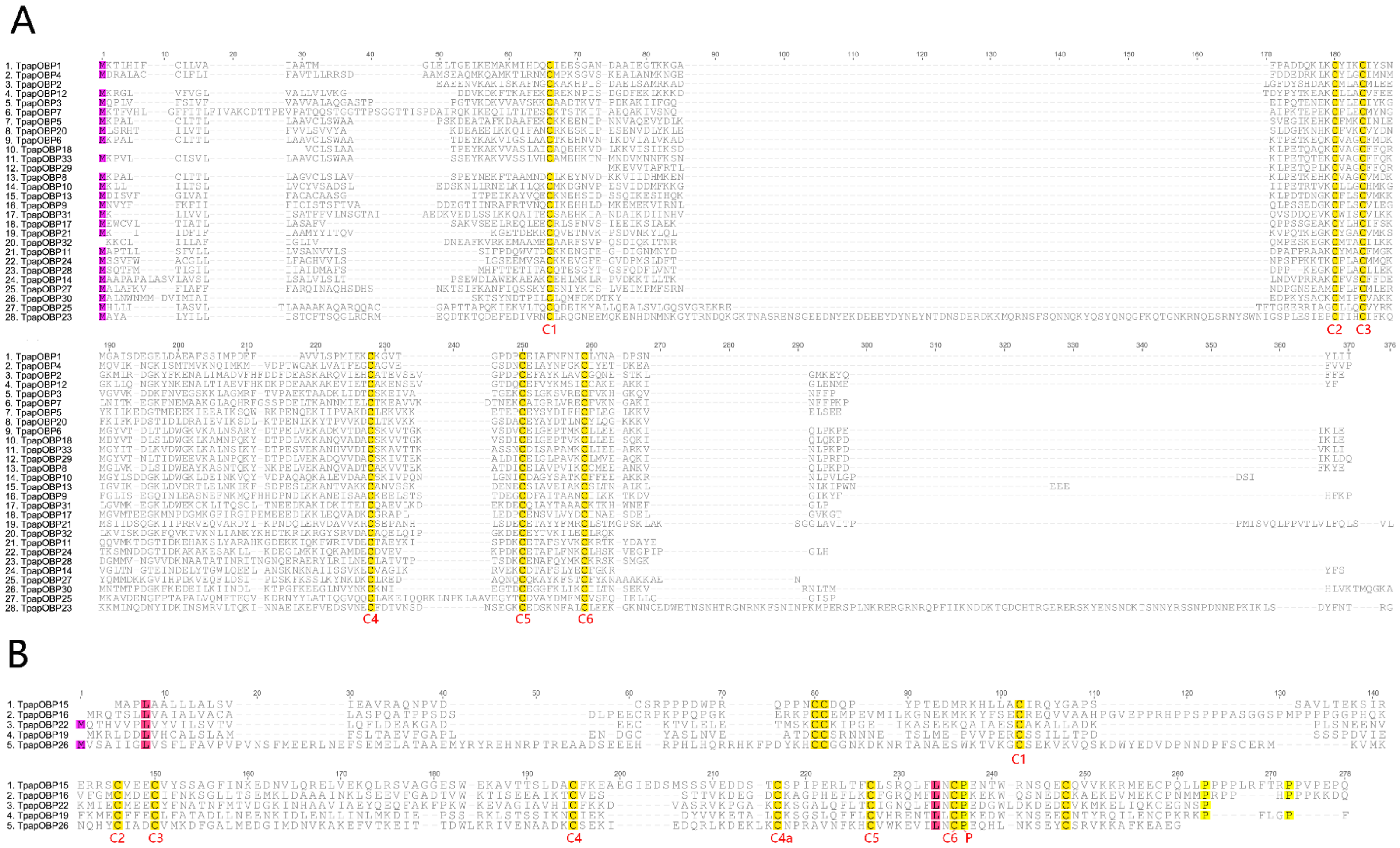

Supplementary Figure S1. Excerpts from the amino acid alignment showing the predicted Classic OBPs (A) and Plus-C OBPs.

Supplementary Table S1. An overview of the sequencing and assembly process.

Item	FA	MA
Read Length	150	150
Total Raw Reads	48,714,852	48,710,580
Total Raw Bases	7,307,227,800	7,306,587,000
Total Clean Reads	48,634,546	48,627,008
Total Clean Reads Ratio (%)	99.84	99.83
Total Clean Bases	7,295,181,900	7,294,051,200
Total Clean Bases Ratio (%)	99.84	99.83
Total Adatper Reads	60,780	65,104
Total Adatper Reads Ratio (%)	0.12	0.13
Total Low Quality Reads	19,526	18,468
Total Low Quality Reads Ratio (%)	0.04	0.04
Clean Reads GC (%)	42.00	39.58
Clean Reads Q20 (%)	96.39	96.09
Clean Reads Q30 (%)	91.25	90.61
**Combined Trinity assembly of the male and female antennal transcriptomes**		
Total Number	74,183	
Min Length	200	
Max length	24,277	
Mean Length	1096	
Unigene N50	2342	
Unigene N90	374	
Unigene GC (%)	34.09	

Supplementary Table S2. Amino acid sequences of *T. papillosa* and other insects used in phylogenetic analyses.

Genes	Insect catalogue	Species	Annotation	Identified Genes	Selected Genes	References
OR	Bug	*Tessaratoma papillosa*	Antennal transcriptome	59	59	This study
	Bug	*Apolygus lucorum*	Antennal transcriptome	110	92	[1]
	Bug	*Halyomorpha halys*	NCBI		25	NCBI
	Planthopper	*Sogatella furcifera*	Antennal transcriptome	63	49	[2]
iGluRs/IRs	Bug	*Tessaratoma papillosa*	Antennal transcriptome	14	14	This study
	Fly	*Drosophila melanogaster*	Genomes	80	80	
	Moth	*Bombyx mori*	Genomes	31	18*	[3]
	Aphid	*Acyrthosiphon pisum*	Genomes	22	22	
OBP	Bug	*Tessaratoma papillosa*	Antennal transcriptome	33	33	This study
	Bug	*Halyomorpha halys*	Antennal transcriptome	30	30	[4]
	Bug	*Lygus lineolaris*	Antennal transcriptome	33	33	[5]
	Bug	*Apolygus lucorum*	Antennal transcriptome	38	38	[6]
	Bug	*Adelphocoris lineolatus*	Antennal transcriptome	14	14	[7]
	Bug	*Adelphocoris suturalis*	Antennal transcriptome	16	16	[8]
	Aphid	*Sitobion avenae*	Antennal transcriptome	13	13	[9]
	Aphid	*Acyrthosiphon pisum*	Genomes	18	17	[10, 11]
	Aphid	*Aphis gossypii*	Antennal transcriptome	9	9	[12]
	Planthopper	*Nilaparvata lugens*	Antennal transcriptome	10	10	[13]
	Planthopper	*Sogatella furcifera*	Transcriptomes	12	9	[14]
	Psyllid	*Diaphorina citri*	Antennal and Abdominal Transcriptomes	9	9	[15]

*antennal IRs

References

1. An, X.K., *et al.* Identification and expression analysis of an olfactory receptor gene family in green plant bug *Apolygus lucorum* (Meyer-Dür). *Sci. Rep.* 6, 37870 (2016).
2. He, M., Zhang, Y.N. & He, P. Molecular Characterization and Differential Expression of an Olfactory Receptor Gene Family in the White-Backed Planthopper *Sogatella furcifera* Based on Transcriptome Analysis. *PLoS One* 10, e140605 (2015).
3. Croset, V. *et al.* Ancient protostome origin of chemosensory ionotropic glutamate receptors and the evolution of insect taste and olfaction. *PLoS Genet.* 6, e1001064 (2010).
4. Paula, D.P. *et al.* Identification and expression profile of odorant-binding proteins in *Halyomorpha halys* (Hemiptera: Pentatomidae). *Insect Mol. Biol.* 25, 580–594 (2016).
5. Hull, J.J., Perera, O.P. & Snodgrass, G.L. Cloning and expression profiling of odorant-binding proteins in the

tarnished plant bug, *Lygus lineolaris*. Insect Mol. Biol. 23, 78-97 (2014).

6. Yuan, H.B., *et al*. Molecular Characterization and Expression Profiling of Odorant-Binding Proteins in *Apolygus lucorum*. *PLoS One* 10, e140562 (2015).
7. Gu, S.H., *et al*. Identification and tissue distribution of odorant binding protein genes in the lucerne plant bug *Adelphocoris lineolatus* (Goeze). *Insect Biochem. Mol. Biol.* 41, 254-263 (2011).
8. Cui, H.H., *et al*. Odorant-binding and chemosensory proteins identified in the antennal transcriptome of *Adelphocoris suturalis* Jakovlev. *Comp. Biochem. Physiol. Part D Genomics Proteomics* (2016).
9. Xue, W., *et al*. Identification and Expression Analysis of Candidate Odorant-Binding Protein and Chemosensory Protein Genes by Antennal Transcriptome of *Sitobion avenae*. *PLoS One* 11, e161839 (2016).
10. Zhou, J.J., *et al*. Genome annotation and comparative analyses of the odorant-binding proteins and chemosensory proteins in the pea aphid *Acyrthosiphon pisum*. *Insect Mol. Biol.* 19 (Suppl. 2), 113-122 (2010).
11. Vieira, F.G. & Rozas, J. Comparative Genomics of the Odorant-Binding and Chemosensory Protein Gene Families across the Arthropoda: Origin and Evolutionary History of the Chemosensory System. *Genome Biol. Evol.* 3, 476-490 (2011).
12. Gu, S., *et al*. Identification and Expression Profiling of Odorant Binding Proteins and Chemosensory Proteins between Two Wingless Morphs and a Winged Morph of the Cotton Aphid *Aphis gossypii* Glover. *PLoS One* 8, e73524 (2013).
13. Zhou, S.S., Sun, Z., Ma, W., Chen, W. &Wang, M.Q. De novo analysis of the *Nilaparvata lugens* (Stal) antenna transcriptome and expression patterns of olfactory genes. *Comp. Biochem. Physiol. Part D Genomics Proteomics* 9 (Part D 9), 31-39 (2014).
14. He, M. & He, P. Molecular characterization, expression profiling, and binding properties of odorant binding protein genes in the whitebacked planthopper, *Sogatella furcifera*. *Comp. Biochem. Physiol. B Biochem. Mol. Biol.* 174, 1-8 (2014).
15. Wu, Z., Zhang, H., Bin, S., Chen, L., Han, Q. & Lin, J. Antennal and Abdominal Transcriptomes Reveal Chemosensory Genes in the Asian Citrus Psyllid, *Diaphorina citri*. *PLoS One* 11, e159372 (2016).

Supplementary Table S3. List of primers used in qPCR.

Gene Name	Forward Primer (5'-3')	Reverse Primer (5'-3')	TM (℃)	Product size (bp)
Reference genes				
TpapUBE4A	GGAGCGGTTGGTTGTGTTAG	TCTCCTTGCTCTGTAGGCTG	59	224
TpapRPL32	AACAGGGTTCCGCAAAGTTC	CCTGAGACGAGCATTGGGAT	59	178
OBPs				
TpapOBP1	GAATCAGGAGCGAACGATGC	TTGAAAGCCAACTCACAGGG	59	245
TpapOBP2	TGCCGAACTAAGTGCAATGAG	GCAAATTCGCATGGATCTGG	59	249
TpapOBP3	CCACTCGTCTTCTCCATCGT	TTCATTCTCGGTCTGTGGGA	59	183
TpapOBP4	GCTTGTTCCTCATCTTCGCA	CTTCGTCGTCAAATTCCCCA	59	168
TpapOBP5	ACCTGCTCTGTGTCTTACCA	CCATTGTCCCGTCTTCTTTCA	59	245
TpapOBP6	CGAGATATGACACCCCGGAG	GCTTGATCTCTGGTTTCGGC	59	168
TpapOBP7	TACCACACCTGAAGTCCCAG	CTTTGCCTGTTCTGCTGTGA	59	166
TpapOBP8	TGGCGGTATCCCCAGAATAT	TTCTCGACCAACTCAGGCTT	59	250
TpapOBP9	GTTGCGGATGATGAAGGGAC	ACCAAATCCCTCCAAGACACA	59	171
TpapOBP10	GCTTTTGATCCTGACTTCGCT	ATCATCGCTCAGGTATCCCA	59	232
TpapOBP11	TGTTCTCCTGCTCGTCTCAG	CACTTGCTGCTTTCCCATGA	59	181
TpapOBP12	GAGAGGGCTGGTCTTCGTAG	AGACACAAGCTAGGAGACACT	59	197
TpapOBP13	ATCTGTCTTCGGGCTTGTGG	GTCCACGTCCAATTTGCCAT	59	232
TpapOBP14	GGCCTGTTGACAAGAAGACG	GCCACACATTCTTTCACCGA	59	205
TpapOBP15	ACGGAAGACATGAGGAAGCA	ACAGCTTTCTCCCAGGACTC	59	224
TpapOBP16	GTTCACCAATGCCACCACC	TTGACTGTGGTGCTGCTTTC	59	223
TpapOBP17	AACCATCGCTACTCTCCTGG	TTCCCTTCCTCGGTCATCAC	59	210
TpapOBP18	TTTGCGCTCAGGAACACAAA	CATCGCCTTCAGTTTTCCCC	59	164
TpapOBP19	CCGCCGACTTGTTGAATGAA	GCAGTGTGTTTTCTCGGTGA	59	207
TpapOBP20	TCAAACCAGATTCCACCATTGA	AAGCATATTCACAAGCGTCAGA	59	150
TpapOBP21	AGAAGGGAAGTGCTATGGGG	CACATTCATCGCTCAGGTGG	59	188
TpapOBP22	AAGTTGCAGGAATCGCAGTG	ATCTAGCCACCCATCTTCCG	59	152
TpapOBP23	GCCTACGCGCTCTACATACT	ACCACTATTTTCTCGTGAGGC	59	228
TpapOBP24	GACGGAACCATAGACAAGGC	AGTGTAAACCTGGGATGGGG	59	190
TpapOBP25	TACGCTCTCCTCCAAGAAGC	CCAAACACTGCTGAACTCCC	59	244
TpapOBP26	TCGGAGAAGGTGAAAGTCCA	ATGCCATCTTCCATCAACGC	59	158
TpapOBP27	GCCCAACATTCTGATCACTCA	ACACATTGCTTCTGAGTTACCA	59	156
TpapOBP28	AAGTGTTTCCTGGCTTGTCT	CACTTGCCCATGGACTTTGA	59	233
TpapOBP29	AACTCCCAGAAACTCAGCCA	AGGCATTTTACAGCAGGCAA	59	232
TpapOBP30	ATGATTCCATGCGTTGCTAAAA	ACCTTCACAATCAGTCCCCT	59	168
TpapOBP31	AAGCAGGCTATCACTGAATGT	ACCTTCCTTCATCACACCCA	59	150
TpapOBP32	CAAACCGACAGATGCCAGAA	TCACATTCATCTTTCCCAGGAA	59	220
TpapOBP33	TACTGTGCCTGTCCGTTCTT	GGCAACACACTTCTCGGTTT	59	185
ORs				
TpapOR1	GATACCTACGATGCGGACCA	TCATTCGGGACAGAGCAGTT	59	199
TpapOR2	CTCTCCCGAATTGAACGCAG	GCCCTGAAAGAAACTGAGGC	59	223
TpapOR3	TATGAAGGTCCGGGAAGCAC	TGGTTTCTTTGTCTGCCTCA	59	213
TpapOR4	GGTTCCATCGCCAAACTGTT	ATGTGCATGTTGTGGGTGAC	59	173
TpapOR5	AATGATTGGTGTCGCATGGG	GATGCCCCAAAAGAACTGCA	59	221
TpapOR6	CGCTTCATCAACCACTACCG	GGTGAGCAGTATCCCACACT	59	249
TpapOR7	TACTGGGACGACAATGAGGG	AGGCTTCTTGACCCTCCAAA	59	216
TpapOR8	CGACTATAAGCGAGAAGGGGT	CCTCCAGTCACCTCGTCAAT	59	180
TpapOR9	ATTGACTACATCGCCCACCA	CCTCCAAGGTAGACACACAGT	59	250
TpapOR10	AGTCAACCACGCTTACAGGA	TATAACACTGTCGCCGCTCT	59	247
TpapOR11	CCGTCCAGCTTATCAACACG	GCCTGAAGATGTCGCTCAAG	59	156
TpapOR12	TGCCAGTTGTCATTGATTGTCT	GAAACCCTTCTGTTCCCACA	59	189
TpapOR13	AGTGTCCTTCATCGTTGGGA	CCACCCTCCTATTGTCGTCA	59	172
TpapOR14	CAACGGTTTAAAGGAGGCCC	GCAGTAGACACAGACCCAGT	59	205
TpapOR15	ATGAGGCAGAACACAAAGCG	TGGACTCCAGCAACGGTATA	59	162
TpapOR16	TGGGCAAATTCTCGCTACCT	TGCGGGGAAATGATGAGGTA	59	166
TpapOR17	ATCCTGTGCCTATGTCCGAG	ACCGAGCCTAACTTCATGGT	59	184
TpapOR18	ACTCCAAGTACATCGGGTCC	GAGTGTGCCGACTACTACCA	59	203
TpapOR19	CGCCTTCTTCGTTCTCATGG	AGGCCGTCATATCCCTTCAG	59	150
TpapOR20	TCATCCGTTCAGTATCTCGCA	GGTCTCACCCTTCCTAGCTC	59	186

Continued

TpapOR21	CGAAACTGACCCGTTGAAGA	GAGCAGCCTCGAAACTCTTT	59	161
TpapOR22	ATTCCAGATCACAAGCGAGC	CAGTCACCGCCAACATACTG	59	181
TpapOR23	GGATCTGTGTGTCCTCGGAA	CACCACCCAGTTCACTTTGG	59	152
TpapOR24	CCCTTTCAACAACGCTGACA	CCGGTGACATCTCCCTCTTT	59	192
TpapOR25	TTCTCTGGAGCTTTGGTGGT	GAAGACGTATGCCCCTCGTA	59	204
TpapOR26	TGGCCCTGGACTATTGGTTT	TCAGCTTCCAGTGTGACCTT	59	175
TpapOR27	GCCTGACATTTGCAAGACCA	CTCAGCATAACCCCTCCGAT	59	210
TpapOR28	CGCCAGGTTGCCATACTTTT	AATTCGCTTGAGTCGATGGC	59	202
TpapOR29	ACATCTGTGCCCTGTTTCAT	AAAAGAATCAGCCAAGCCCG	59	170
TpapOR30	TCACCCTCCAACTAACAGCT	CCAACCAACAAAGTACGGCA	59	250
TpapOR31	CGTCTTGAGGTTCTTGGGGA	CTGTTTGTGCATCTGGAGCA	59	151
TpapOR32	GGCAAACCTGTTCTCCGTTT	CACCTCCTCATTCCATTGCG	59	177
TpapOR33	GTCTCGATGTTCTGCGTTGT	GGTCTCACCCTTCCTAGCTC	59	150
TpapOR34	ATTGTGGAGGGAGATGGGTG	AGTGGAGCAAGCGAAGTAGT	59	235
TpapOR35	GCCACATCCTCTTTACCGTC	ACAGCAGACCAGAAACCAGT	59	249
TpapOR36	CAGCGCATTGTTCCTAGGAC	CGCACATGTAGTTGGAGGTG	59	218
TpapOR37	CCTTCGAGGCGGACAGTATT	CCTCGGTTCCAAGTTCCTCA	59	176
TpapOR38	CTTATCACGGCCAGTTTGCA	TACTTCTTGTCGGCCAGGTT	59	194
TpapOR39	TCTGGCCGTGTATGTCTGTT	GTTCGCCCGCATAACTCAAG	59	212
TpapOR40	GTAGTGGACCCGGGGATATC	CCGAGCACAGCGAATTGTAA	59	248
TpapOR41	GGCTTTCCACTTCGTCCTTC	GCAATCTTCCGATGTAGGCG	59	171
TpapOR42	TGTCGTATGAGGCCACAGAA	GTGAGGGTTTGCGGTTTCAA	59	175
TpapOR43	CAGGGGTATCATGGGTAGGC	AGGCAGTTGAAAAGGAGGGA	59	153
TpapOR44	AATCGGGAATCAGCTAACGC	AATCCTGCGAACGTACATGC	59	176
TpapOR45	GACAGCAGCGAGTTCTTGAG	ATGCGATGATGATGCGGTTC	59	226
TpapOR46	GTTCTACCGACCACTTTGCC	AGTGGATTCGCACTGTCAGA	59	218
TpapOR47	TCCTCCCATTCTAGCTTCCA	TCCGTCTTCCATCATTCTCCT	59	163
TpapOR48	CTCGCTGCAATGTTTGTCCT	TGGATGTCTGCAAAACCAACA	59	175
TpapOR49	AGTTCGGCTGCGTCTTCTAT	GCGATGAACACAGTGAGGAG	59	175
TpapOR50	GGTACTTGTCCGAATCGCAG	AGTTGGTTGCCGATGGTTTC	59	196
TpapOR51	CAAAACCCTGTTCAGCACGA	ATACTCGGACCTGATGCCTG	59	187
TpapOR52	ATTGACTACATCGCCCACCA	ACACAGTACCCAAGCACAGT	59	236
TpapOR53	TCGATGCCTCCAAGAACAGT	CGACGACATGCAAGCAGAAT	59	204
TpapOR54	ATCTGAAATGGTTGTCGGCG	CCCTTGAATTGCATCGTCGT	59	245
TpapOR55	GACTTGCCATGTGAGGGTTT	TGTTTGATGATTGCTAGCTCTGT	59	176
TpapOR56	AGCGAGGGAGATAGTGATGC	GGATTCAGCGTTTCCCCAAA	59	212
TpapOR57	CGTCTATGTGTGGGAGGTGA	ATCACGCCGCTTCTGATTTC	59	237
TpapOR58	TATGCCCACTTTGTCGTCCT	CTGTATCCAGCGCCAATGTC	59	214
TpapOR 59	ACTTGGCCTCCTTTCATCGA	TCAGAGCGTCCTTTGTTCCT	59	203
Antenal IRs				
TpapIR8a	AGACATTGGTATCACGCCCT	GGACAGTCACCCGAAGAGAT	59	155
TpapIR25a	TATCATGCCACTGTCACGGT	AGCGTCCACTTTAACTCCCA	59	150
TpapIR76b	TTTGGGAAAGTTGGCCTCAG	CCAAAAGACCACTGCCAGTC	59	164
TpapIR92a	TGACGTGTGGATAGCGTTCT	GCTCTCATGCTGCTGTCTTC	59	150
TpapIR93a	TACAGCAGGGAGGAGGAGTA	GAAGGGTGGAATGTTCTGGC	59	216
TpapIR75d.1	CTGACCTCACGAACAGTCCT	AAGCAAACAACCCAGATCGG	59	186
TpapIR75d.2	CTCCTCACCCTCCTAATCGG	ACGGAGGTTACAGTCTCAGC	59	176
TpapIR84a	TTTCACCTTCACCGCCTCTA	AGTCCAGGTTCCGTTATGCA	59	217
